# Liquid Biopsies: Applications for Cancer Diagnosis and Monitoring

**DOI:** 10.3390/genes12030349

**Published:** 2021-02-27

**Authors:** Ivana Martins, Ilda Patrícia Ribeiro, Joana Jorge, Ana Cristina Gonçalves, Ana Bela Sarmento-Ribeiro, Joana Barbosa Melo, Isabel Marques Carreira

**Affiliations:** 1 Cytogenetics and Genomics Laboratory, Faculty of Medicine University of Coimbra, Institute of Cellular and Molecular Biology, University of Coimbra, 3004-531 Coimbra, Portugal; ivanamrts@gmail.com (I.M.); ildaribeiro.patricia@gmail.com (I.P.R.); mmelo@fmed.uc.pt (J.B.M.); 2 Center of Investigation on Environment Genetics and Oncobiology (CIMAGO), Faculty of Medicine University of Coimbra, Coimbra Institute for Clinical and Biomedical Research (iCBR), University of Coimbra, 3004-531 Coimbra, Portugal; joanaverdasca@gmail.com (J.J.); acgoncalves@fmed.uc.pt (A.C.G.); absarmento@fmed.uc.pt (A.B.S.-R.); 3 Center for Innovative Biomedicine and Biotechnology (CIBB), University of Coimbra, 3004-531 Coimbra, Portugal; 4 Clinical Academic Center of Coimbra (CACC), 3004-531 Coimbra, Portugal; 5 Laboratory of Oncobiology and Haematology and University Clinic of Haematology, Faculty of Medicine, University of Coimbra, 3004-531 Coimbra, Portugal; 6 Clinical Haematology Department, Coimbra University Hospital Centre (CHUC), 3004-531 Coimbra, Portugal

**Keywords:** cancer, liquid biopsies, diagnosis, prognosis, monitoring, cell-free DNA, circulating tumor cells, precision medicine

## Abstract

The minimally—or non-invasive detection of circulating tumor-derived components in biofluids, such as blood, liquid biopsy is a revolutionary approach with significant potential for the management of cancer. Genomic and transcriptomic alterations can be accurately detected through liquid biopsies, which provide a more comprehensive characterization of the heterogeneous tumor profile than tissue biopsies alone. Liquid biopsies could assist diagnosis, prognosis, and treatment selection, and hold great potential to complement current surveilling strategies to monitor disease evolution and treatment response in real-time. In particular, these are able to detect minimal residual disease, to predict progression, and to identify mechanisms of resistance, allowing to re-orient treatment strategies in a timelier manner. In this review we gathered current knowledge regarding the role and potential of liquid biopsies for the diagnosis and follow-up of cancer patients. The presented findings emphasize the strengths of liquid biopsies, revealing their chance of improving the diagnosis and monitoring of several tumor types in the near future. However, despite growing evidence supporting their value as a management tool in oncology, some limitations still need to be overcome for their implementation in the routine clinical setting.

## 1. Introduction

As the cancer burden keeps on growing globally, continuous efforts are made in order to improve the diagnosis and the management of this disease. Early diagnosis remains one of the main challenges in cancer care [1]. Thus, the development of screening and early detection tests, along with the establishment of efficient monitoring methods, are of great importance to improve the efficacy of therapies and to reduce cancer mortality [2]. In this sense, precision medicine has gained particular attention in the oncology field [3]. Molecular profiling can be applied to gain insight into the alterations underlying tumorigenesis [4], contributing to the identification of diagnostic and prognostic biomarkers, and to the selection of treatments considering individual variability.

Although tissue biopsies are currently the gold standard for tumor profiling, this method presents many limitations: besides being invasive, risky, and, for some anatomical locations, not easily obtained [5], it provides a limited picture of the tumor profile. In fact, tumors are heterogeneous entities, containing various subpopulations of cells that harbor different alterations. In addition, tumor cells undergo dynamic genetic and epigenetic changes through time (due to therapeutic stress [6], for example), resulting in further tumoral heterogeneity and in discrepancies between primary and metastatic lesions [5]. Thus, the spatially and temporally limited tissue biopsies fail to represent the overall tumor profile, to capture alterations from different sites and, consequently, to monitor disease progression [7]. 

Given this, in the recent years, research in oncology has focused on liquid biopsies, which rely on the detection of cancer-derived components, including circulating tumor cells (CTCs) [5,8,9,10,11,12,13,14,15,16], circulating tumor DNA (ctDNA) [3,4,6,7,12,16,17,18,19,20,21,22,23,24,25,26,27,28,29,30,31,32,33,34,35,36,37,38,39,40,41,42,43,44,45,46,47,48,49,50,51,52,53,54,55,56,57,58,59,60], RNA [10,61,62,63,64,65], extracellular vesicles (EVs) [10,66], and tumor educated platelets (TEPs) [67], in the biofluids of patients, providing genomic [68,69], epigenetic [70,71], transcriptomic, and proteomic [72] information about tumors and metastatic sites. The use of liquid biopsies as a clinical tool will improve cancer screening [2], diagnosis [15,17,19,53,58,61,62] and prognosis [4,13,25,26,29,48,73], ameliorate the classification of more heterogeneous entities, and perform a tighter patient monitorization [56,64,65,74], assessing treatment response [3,14,23,52,63,66,75] and detecting treatment-resistant clones [39,42,51]. All this can be achieved by the introduction of this minimally invasive procedure that can be repeated several times throughout the disease progression without arm for the patients. Moreover, liquid biopsies provide a broader genetic characterization of the tumor reflecting its heterogeneity [9,36,40,49] and possibly identify disseminating aggressive clones.

In this review we sought to collect current evidence regarding the potential of liquid biopsies for the diagnosis and follow-up of cancer patients, as well as the advantages and limitations of this approach.

## 2. Materials and Methods

### 2.1. Search Strategy

In order to retrieve recent findings concerning the utility of liquid biopsies for cancer diagnosis and/or monitoring, searches for studies published in the last five years (2015–2020) were conducted on PubMed and Google Scholar. In PubMed, the search terms “liquid biopsy”, “cancer”, “diagnosis”, and “monitoring” were used to search titles and/or abstracts of human studies, in the cancer field, and written in English. The same terms were used to screen study titles in Google Scholar. 

After removal of duplicates, the titles and abstracts of the retrieved studies were screened for their suitability. Abstracts revealing clear exclusion criteria were removed. The remaining studies were reviewed for eligibility based on the full text.

### 2.2. Eligibility Criteria 

Only original articles that enrolled more than 25 adult cancer patients were included. Nonhuman studies, studies regarding pediatric cancers or studies that did not address the applications of liquid biopsies for cancer diagnosis or follow-up were excluded. Reviews, systematic reviews and meta-analyses were also excluded.

## 3. Results

**Figure 1:**
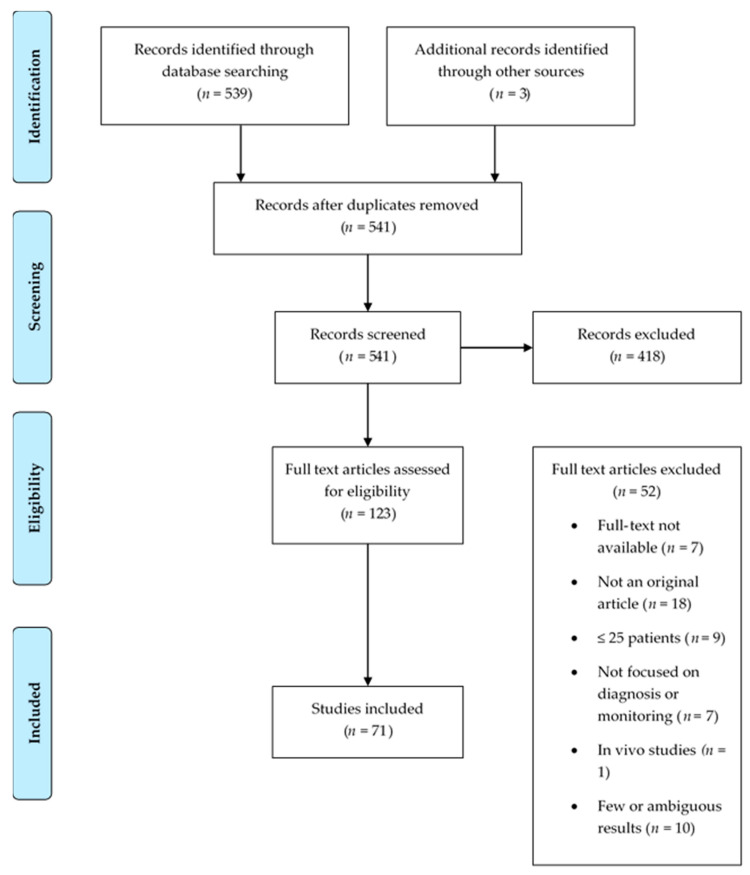
PRISMA flow diagram of the search strategy to retrieve studies included in the review.

As seen in Figure 1, our database search yielded a total of 539 results. An additional study was identified by checking the references of the retrieved articles. After removal of duplicates, a total of 539 studies were screened by their title and abstract, and 123 remained. The full text of these studies was examined to determine their eligibility, and 52 articles were excluded. Finally, a total of 71 studies were included in this review.

## 4. Discussion

The review of the selected articles revealed that liquid biopsies are a topic of growing interest in cancer research, given their particularly enticing characteristics and large number of potential clinical applications. This type of biopsies hold promise to improve several aspects of cancer management, including tumor profiling, with implications for diagnosis and treatment selection, prognosis, and long-term monitoring, paving the way towards precision oncology and better patient outcomes.

The main findings of the retrieved studies were summarized in Table 1.

### 4.1. Liquid Biopsies for Diagnosis and Tumor Profiling

Currently, cell-free DNA (cfDNA) is one of the most studied analytes in liquid biopsies. Both the quantity [17,21,22,26,39,47,52] and the integrity [21,22,26] of cfDNA in circulation have shown the ability to distinguish cancer patients from healthy individuals. The total levels of cfDNA tend to be higher in cancer patients than in healthy subjects [17,21,22,26,39,47,52], and seem to increase with stage [17] and metastasis [55]. The increased concentration of cfDNA in these patients is thought to reflect the additional release of genetic material from tumor cells, but it could also be a result of the defective clearance of circulating DNA by phagocites [79]. However, high cfDNA levels are not specific of cancer and have been identified in other pathological and non-pathological conditions, including exercise, trauma and surgery [22], which might hamper their direct application for cancer diagnosis. Regarding integrity, cancer patients seem to display higher cfDNA fragmentation (<100 bp) than healthy controls [22,26], although a study in thyroid carcinoma revealed the contrary [21], once more disclosing that this type of analyses still lack the sensitivity and specificity needed for diagnosis. 

Nevertheless, through the analysis of tumor-specific alterations, including single nucleotide variants (SNVs), insertions, deletions, copy number variations (CNVs) [40], and methylation alterations [16,57,59], one can identify tumor-derived DNA—ctDNA, among the total pool of cfDNA, providing a much more accurate form of cancer genotyping and, consequently, of diagnosis. Importantly, these (epi)genetic alterations seem to be highly concordant in blood ctDNA and in corresponding tumor tissues in a variety of cancers, including lung [17,29,42], breast [35], colorectal [17,44,50,52], pancreatic [32], liver [57], esophageal [17], gastric [6,43], and ovarian [17] cancers. In this sense, these minimally-invasive and less risky liquid biopsies [57] could be used as an alternative to tissue biopsies in cases in which the latter cannot be performed [24,39,49] or when these do not gather enough high-quality DNA [17,39]. In fact, since 2016, non-small cell lung cancer (NSCLC) patients who are unable to provide tumor specimens can be tested for *EGFR* mutations in plasma, using the U.S. Food and Drug Administration (FDA)-approved cobas EGFR Mutation Test v2 [80]. Since then, several other liquid biopsy-based tests have been approved by this agency: plasma samples are now being used to detect specific gene mutations and rearrangements in patients with ovarian, lung, breast, and metastatic castration resistance prostate cancers. These tests are mainly used as companion diagnostic tests to identify patients who are eligible for targeted treatments [81].

However, the concordance of alterations found in ctDNA and tumor tissues varies not only according to the type of cancer [19], since different tumors have different probabilities of shedding DNA into the bloodstream [20], but also depending on the stage of the disease [17,36]. In fact, cfDNA levels tend to be lower in earlier stages [17,18], suggesting a limited role of liquid biopsies for the early detection of cancer [17,48], although increased technological sensitivity might help overcome these problems [17]. Even though the use of liquid biopsies for cancer screening is still largely undeveloped, a liquid biopsy test based on the detection of abnormal methylation of the *SEPTIN9* gene in blood has already been approved to screen for colorectal cancer (CRC) [82]. In addition, other blood tests that use ctDNA to screen for several cancer types, including ovarian, liver, stomach, pancreatic and esophageal, which, to date, do not have screening tests available, are currently being studied [83].

In addition to accurately representing the tumor profile, ctDNA also captures tumor heterogeneity [40,44,53]. This is particularly relevant in metastatic disease, in which multiple tissue biopsies might not be viable and are associated with increased cost and risks to the patients [40]. Correctly profiling all tumor sites is especially important for the detection of actionable mutations and, as mentioned above, for the selection of patients that might benefit from targeted therapies [19,44]. Still, ctDNA analysis might be less sensitive to detect some alterations that are found in tumor tissues [19,41], since these are diluted in a background of germline DNA [12,20] and some might be present in small subsets of cells and, thus, exist in lower quantities in circulation [6,60]. Therefore, currently, liquid biopsies seem to have a more complementary rather than alternative role to tissue biopsies for diagnostic and profiling purposes.

Besides blood, other biofluids, such as urine [7,23,28,37,39,47,52], cerebrospinal fluid (CSF) [60], and gastric washes [54], have been shown to harbor ctDNA. Depending on the type of cancer, tumors might more closely contact with different fluids that, consequently, might contain higher ctDNA concentrations than blood [23]. For example, urinary ctDNA has been detected in bladder [23,37,58] and other urothelial cancers [23]. Moreover, transrenal DNA, resulting from the clearance of bloodstream cfDNA by the kidneys, has also been detected in non-urological tumors, such as NSCLC [28] and CRC [39,52]. Notably, urinary ctDNA also presents cancer-specific mutations [23], CNVs and methylation alterations [58] that are highly concordant with the ones found in tumor tissues [39,47,52]. Given this, the use of urine for liquid biopsies is particularly promising considering that its collection is totally non-invasive, resulting in improved patient compliance for serial sampling for diagnosis or follow-up [39]. Nevertheless, although there seems to be a positive correlation between plasma and urine cfDNA levels [37,52], mutation detection in urine is usually limited in comparison to blood [39]. Regarding CSF, this is a specially relevant source of information in brain tumors, such as gliomas [19,60], as an alternative to surgical tissue biopsies. Importantly, CSF-derived ctDNA has also been shown to display tumor-concordant mutations, CNVs, and structural rearrangements [60]. 

In addition to ctDNA, other components with diagnostic potential can be investigated in liquid biopsies, such as messenger RNA (mRNA) [4,62] and micro-RNAs (miRNAs) [61,64]. For instance, Malczewska et al. evaluated gene expression in the blood of patients with bronchopulmonary carcinoid (BPC) tumors and revealed that the levels of target transcripts were significantly increased in comparison to healthy controls and enabled to distinguish metastatic and localized disease. Importantly, gene expression was highly correlated in tumor tissue and blood [4]. Similarly, the levels of several miRNAs are often altered in cancer patients, allowing to determine miRNA signatures with diagnostic and prognostic potential [61,65]. Tumors and their microenvironment release miRNAs that exist in the bloodstream in ribonucleoprotein complexes or incorporated into EVs [65]. In particular, circulating miRNA profiles seem to be concordant with the ones of tumor tissues [64]. However, EV-incorporated miRNAs seem to represent only a small fraction of the miRNAs present in circulation and to have distinct diagnostic performance [61].

The DNA in exosomes (exoDNA) can also be a valuable source of information, given that it is less prone to degradation than cfDNA and released from living cells, thus probably reflecting tumor-driving alterations more accurately than ctDNA that is derived from apoptotic and necrotic cells [32]. Contrarily to exosomes, that are derived from intracellular compartments, tumor-associated microparticles (taMPs) are membrane-derived vesicles that display cell surface markers from their source. As so, the expression of the cancer markers epithelial cell adhesion molecule (EpCAM) and CD147 in taMPs was shown to be specific of cancer patients, which could potentially be used for diagnostic purposes. In addition, these double-positive taMPs significantly correlated with tumor burden in CRC [66].

Blood platelets are local and systemic responders that act during carcinogenesis and metastasis [78]. Platelets are able to directly ingest circulating mRNA and tumor-associated proteins released by tumor cells as well as undergo splice events in response to signals released by cancer cells and the tumor microenvironment [84]. Platelets exposed to tumor-induced education present an altered platelet behavior [77,78]. These platelets (TEPs) have been shown to be highly specific and are able to predict and facilitate early screening of NSCLC [77], predict and discriminate patients with primary glioblastoma from patients with brain metastases and with neuro-inflammatory conditions [78] and predict treatment outcome after therapy [76].

Lastly, the number of CTCs cells that detach from primary and metastatic tumor sites [5], seems to increase with stage and disseminated metastasis [15], revealing its potential as a diagnostic and prognostic marker. Although most CTC isolation systems, including the CellSearch platform, rely on the detection of cell surface markers, such as EpCAM and cytokeratins, important subpopulations of CTCs that do not express these markers or that have undergone epithelial-to-mesenchymal transition (EMT) might be missed using these methods [5,8,67]. In this sense, efforts are currently being made to integrate the isolation of such subsets of cells, for example, through multiparametric analysis [67] or detection of additional relevant (e.g., mesenchymal [8]) markers [5,15]. In addition to the identification and quantification of CTCs, their molecular characterization provides relevant information regarding mechanisms of CTC release and tumor biology [8]. Particularly, methylation status has been shown to be highly concordant in CTCs and in paired plasma ctDNA, suggesting a common source of this DNA [16]. Furthermore, CTCs’ (epi)genetic profiles mirror the ones of the tumors [12,16] and could also be used for targeted treatment selection [11,15]. 

Besides having potential to aid in diagnosis and tumor profiling, liquid biopsies seem to have prognostic value. Firstly, studies have shown that ctDNA detection positively correlates with tumor size [6,18,20,32], informing about disease burden. Secondly, high ctDNA levels at baseline have been correlated with lower survival [17,24,32,45,46] and poorer outcomes [24,60]. Similarly, the number of CTCs also seems to increase with tumor size and to be a predictor of poor survival [9,67]. 

In addition to ctDNA, exoDNA has also been shown to have prognostic value, with *KRAS* mutant allele fraction (MAF) being associated with progression-free survival (PFS) and overall-survival (OS) in pancreatic cancer [32]. Further, tumor-derived platelets biomarkers, as *KLK3, FOLH1*, and *NPY*, enable prediction after abiraterone therapy in castration resistant prostate cancer (CRPC) and *KLK2*, *KLK3*, and *FOLH1* were associated with short OS [67]. 

### 4.2. Liquid Biopsies for the Follow-up of Cancer Patients

A particularly promising application of liquid biopsies concerns its use to monitor disease evolution and treatment response in cancer patients. In fact, the non- or minimally-invasive nature of these biopsies make them a favorable alternative for long-term follow-up [52]. Contrarily, multiple invasive tissue biopsies are not always feasible and, more importantly, these often miss alterations found in sites other than the primary tumor that might influence therapy response and efficacy [46]. In addition, imaging techniques that are often used to monitor cancer patients undergoing treatment present many limitations. For example, computed tomography (CT) scans expose the patients to radiation, are costly [48], have low sensitivity to detect small lesions [3,48] and do not provide information regarding genetic changes induced by treatment [31].

On the other hand, the longitudinal collection of biofluids allows for the monitoring of the disease, assessment of treatment response, and identification of mechanisms of resistance [48,53]. Importantly, given that cfDNA has a short half-life [85,86,87], liquid biopsies allow for the follow-up of cancer patients in real-time. For instance, ctDNA analysis has been successfully used to detect resistant mutations in genes such as *EGFR* [29,47], *ERBB2* [43], *PIK3CA,* and *RAS* [28,50], in various cancers including NSCLC [28,29,47], CRC [50], and gastric cancer [43]. Moreover, monitoring the dynamics of ctDNA alterations is useful to predict response to treatment and clinical outcome [3,6,29,37,39,41,42,45,46,49], and allows to reorient treatment regimens in a more timely manner [25,59]. As a matter of fact, a ctDNA decrease after treatment has been associated with lower risk of progression [3,41,42] and longer survival [42], while persistent or increased ctDNA levels have been associated with progression [3,28,37,42,45,49,55], relapse [33,35,37], and decreased survival [35]. In particular, a promising study has shown that it might be possible to quantitatively predict the time needed for disease progression by combining longitudinal profiling of cfDNA and mathematical modeling [44]. 

Notably, alterations in ctDNA seem to reflect changes in tumor burden in response to treatment, as confirmed by imaging [27,34,35,36,42,48,50,55,59]. Moreover, several studies have proven that ctDNA is able to predict response or resistance to therapy, progression, and relapse several weeks or even months before conventional imaging techniques [3,31,33,35,42,48,50,75]. Given this, ctDNA could be used as first tier test to better assess response in patients undergoing treatment [31,34,48,53,59].

In addition to ctDNA, both the count and characterization of CTCs have been shown to have predictive value [9] and to be useful to monitor therapy response and the dynamic progression of tumors [88]. Given that these cells are closely involved in the metastatic process, monitoring them might help predict the patients’ post-treatment outcomes and risk of metastasis [88,89]. A decline in the number of CTCs after treatment could be related with a better prognosis [89,90]. As a matter of fact, some studies have shown that the number of CTCs tends to decrease in response to therapy [15,90], whereas a postoperative increase in CTC number and in the percentage of mesenchymal CTCs has been shown to precede recurrence [8]. However, a better understanding of the patterns of CTC shedding and clearance is imperative for a correct interpretation of CTC fluctuations [91].

Additionally, similarly to ctDNA, CTCs also seem to predict therapy response more efficiently than imaging techniques. In fact, a study using a gene expression panel to detect CTCs was able to identify metastatic CRC (mCRC) patients that were not responding to therapy, which was unnoticed by CT scans. Besides demonstrating a higher sensitivity than CT, CTC analysis also identified non-responders presenting lower PFS and OS earlier than this imaging technique [9].

Furthermore, the characterization of CTCs during treatment also allows to detect genetic alterations or gene expression changes related to resistance [5,12]. Remarkably, a study has revealed that the detection of the *EGFR* T790M resistant mutation in patients undergoing treatment with tyrosine kinase inhibitors (TKIs) occurred earlier in CTC than in ctDNA analysis [12]. In addition, as proposed by Girotti et al., xenografts established from patients’ CTCs could be used to select second-line personalized therapeutic options [75]. 

Transcriptomic data with monitoring potential can also be investigated in EVs or in circulating RNA. Interestingly, EVs seem to present different mRNA profiles than CTCs and, although the expression of various transcripts might be associated with progression or response to therapy, depending on the source, EVs or CTCs, the same transcript might be associated with different outcomes, revealing the need of exploring which is the most relevant monitoring marker [10]. In addition, Malczewska et al. have shown that blood mRNA levels allowed for the identification of patients with residual disease and for the differentiation of progressive from stable disease in lung neuroendocrine tumors [4]. Assessing the levels of circulating miRNAs might also contribute to monitor disease, as revealed by the decrease of certain miRNAs after management of prostate cancer (PCa) [63] or hepatocellular carcinoma (HCC) [64]. Importantly, significantly higher levels of miRNA-224 were detected in HCC patients with residual tumors, whereas conventional markers and magnetic resonance imaging (MRI) could not distinguish residual disease [64]. Moreover, a study in lung cancer demonstrated that a circulating miRNA signature was able to stratify patients according to their risk of relapse, which adds prognostic value and eventually allows to identify patients that might benefit from adjuvant therapies, for example [65].

The levels of the previously mentioned EpCAM(+) taMPs might also serve as a monitoring tool, as revealed by their decrease after surgical removal of CRC [66]. Another way of assessing successful surgical resection, relying on the evaluation of postoperative Apo10- and transketolase like 1 (TKTL1)-expressing monocytes, has been tested in oral squamous cell carcinoma (OSCC) [74].

Lastly, liquid biopsies have also been used to detect and monitor circulating cell-free Epstein-Barr virus (cfEBV) DNA, for example, in EBV-associated gastric carcinoma (EBVaGC) [51] and nasopharyngeal carcinoma [38]. Monitoring the dynamics of cfEBV DNA in EBVaGC revealed that the levels decrease significantly after treatment, but increased in a patient that developed recurrence [51]. Distinct patterns of cfEBV DNA clearance associated to treatment sensitivity have also been identified in nasopharyngeal carcinoma, demonstrating its prognostic value and usefulness for treatment adaptation. Similarly to what has been depicted in ctDNA studies, the persistence of cfEBV DNA after treatment was associated with worse survival. In addition, the levels of cfEBV at baseline were also associated with the likelihood of treatment response or resistance [38].

Overall, this data reveals that liquid biopsies are useful and advantageous to monitor cancer patients, providing early information regarding disease evolution and treatment efficacy, and allowing to reorient treatment strategies in time. In particular, several studies indicate that the use of liquid biopsies as a complement to current surveilling strategies, such as tissue biopsies and imaging, could substantialy improve the detection of resistance, residual disease and relapse.

Recently, the use of liquid biopsies to predict and to assess response to cancer immunotherapy has emerged as a new area of investigation, which is explored in more detail below.

### 4.3. Liquid Biopsies in the Immuno-Oncology Field

Liquid biopsies have gained particular attention in the immuno-oncology field lately, with several ongoing clinical trials and studies focusing on this subject. The use of programmed death 1 (PD-1)/programmed death ligand 1 (PD-L1) or cytotoxic T-lymphocyte-associated antigen 4 (CTLA-4) immune checkpoint inhibitors (ICIs) has become a standard in the treatment of various cancers, including NSCLC [13,14,56,73], with significant clinical benefits.

Currently, one of the most recognized predictors of response to PD-1/PDL-1 ICIs is PD-L1 expression in tumor tissues [73]. However, not all patients respond to this type of treatment, including some patients with PD-L1(+) tumors. In addition, a subset of PD-L1(−) patients seem to benefit from ICI treatment. This could be, at least in part, due to the heterogeneous PD-L1 expression that is not always captured by tissue biopsies [13]. Thus, new biomarkers to predict and monitor ICI treatment response and outcomes are needed [56]. In this regard, liquid biopsies could be a more appropriate way to identify and track these markers. Given this, studies have investigated the prognostic potential of PD-L1 expression in CTCs [13] and in populations of nucleated blood cells [73]. A study in NSCLC has shown that CTCs were most frequently PD-L1(+) than tumor tissues, which could be a result of tumor heterogeneity or insufficient tissue sampling. A higher number of CTCs at baseline was associated with worse outcomes in patients treated with ICIs, probably on account of underlying extended disease. In addition, although PD-L1(+) CTCs did not correlate with outcomes, all patients with progressive disease presented PD-L1(+) cells and these were observed in higher number at baseline in non-responders, suggesting a possible mechanism of resistance involving this cell subpopulation [13]. Another study has revealed that a higher quantity of PD-L1-expressing peripheral circulating cells was associated with worse survival [73]. Overall, these studies suggest that the detection and monitoring of PD-L1(+) cells might be useful for prognosis and early assessment of ICI treatment response.

Besides PD-L1 expression, serial monitoring of CTC-derived transcripts has also been shown to be predictive of clinical outcome in melanoma patients undergoing ICI treatment [14]. In addition, the evaluation of dynamic CNVs in cfDNA could serve as an early indicator of immunotherapy response or progression [56].

Further studies should evaluate the relationship between PD-L1 expressing CTCs and efficacy of ICI therapies and explore the potential of other liquid biomarkers to predict response and to identify patients eligible for immunotherapy.

### 4.4. Advantages and Disadvantages of Liquid Biopsies

The described evidence revealed that liquid biopsies provide a minimally-invasive way of representing the heterogenous tumor profile [19] at baseline and/or during follow-up [12,19,39], improving patient care without the limitations and risks associated with tissue biopsies. The main clinical applications and limitations of each analyte found in liquid biopsies are summarized in Table 2.

The use of liquid biopsies for follow-up of cancer patients is one of its most investigated applications and seems to be the one that is closer to a broad implementation in the clinical setting. On the other hand, the use of liquid biopsies for screening and early cancer detection remains challenging [40], mainly because analytes, such as CTCs [2] and ctDNA [17,48], are often below optimal levels for analysis, particularly in early-stage patients. A potential solution to improve the sensitivity of cancer detection would be to evaluate multiple circulating analytes (e.g., ctDNA, RNA, proteins [94], and metabolites [95]), however, the specificity and cost-effectiveness of such tests would probably be compromised [2]. In fact, the low specificity of liquid biopsy tests is a concern, given that their use for cancer screening would result in the detection of high numbers of false positives, making healthy individuals undergo unnecessary invasive procedures and distress. For instance, alterations resulting from clonal hematopoiesis in peripheral blood cells can also be found in cfDNA of individuals that do not have cancer [23,53], which, if not carefully analysed, may lead to less correct diagnosis. Despite these challenges, the non-invasive screening and early detection of cancer is still one of the most attractive and awaited applications of liquid biopsies, since it could substantially improve treatment efficacy and patient survival [2,96], especially for cancer types lacking screening tools and that are often diagnosed in advanced stages [96]. Hopefully, the technological advances and growing interest in this topic will result in more evidence regarding the value of liquid biopsies as a tool for cancer screening and diagnosis in the coming years.

Ensuring that cfDNA samples are of sufficient quantity and quality is crucial for the success of downstream applications. For that purpose, contamination of samples with genomic DNA must be prevented, for example, using white blood cell stabilizers. The isolation of cfDNA from plasma samples instead of serum is also preferable, given that it avoids the release of cellular DNA from lysing cells during the clotting process. In addition, due to low concentrations, extraction methods must guarantee high yields of cfDNA [92]. The low quantities and small fraction of ctDNA in circulation require the use of highly sensitive detection techniques, such as droplet digital Polymerase Chain Reaction (ddPCR), Next Generation Sequencing (NGS) [41] or BEAMing (beads, emulsion, amplification and magnetics) [12]. However, due to temporal heterogeneity, targeted sequencing limits treatment response monitoring and detection of resistance mutations [36], evidencing the need to use broader panels to analyze ctDNA during follow-up [41], which might compromise sensitivity of detection [44]. ctDNA profiling is still complex and expensive to apply in clinical routine [30,40]. Additionally, the isolation of CTCs, which are extremely rare in circulation, is also difficult [5,12,16] and costly [30]. Instead, the quantification of total cfDNA could be used as a simple and cheap alternative to predict disease response and outcomes, as revealed by a study in metastatic breast cancer (mBC) [30], although its diagnostic value is limited, as explained previously.

In conclusion, despite still presenting some limitations, liquid biopsies hold great potential to improve clinical care in oncology. In particular, this type of biopsy offers opportunities to improve the surveillance of cancer patients during treatment and might be helpful to supplement current diagnosis and tumor profiling strategies in the near future.

## Figures and Tables

**Table 1:** Overview of the studies included in the review.

Author, Year (Trial Code, If Applicable)	Number of Patients and Type of Cancer	Type of Biofluid and Analyte	Main Findings
Schwaederle, M. et al., 2015 [19]	171 patients, including lung (*n* = 40) and breast (*n* = 40) cancers, glioblastoma (*n* = 33), and others	Plasma circulating tumor DNA (ctDNA)	ctDNA alterations, most of which were potentially targetable by approved drugs, were detectable in 65% of various cancers and in 27% of glioblastomas.
Shoda, K. et al., 2015 [25]	77 gastric cancer patients	Plasma ctDNA	*HER2* amplification can be detected in plasma which might have utility in predicting treatment efficacy in gastric cancer.
Sestini, S et al., 2015 [65]	84 lung cancer patients	Plasma micro-RNA (miRNA)	The studied 24 plasma miRNA signature seems to have utility as a prognostic and monitoring tool in lung cancer.
Girotti, M.R. et al., 2015 [75]	214 melanoma patients	Blood circulating tumor cells (CTCs) and plasma ctDNA	Longitudinal ctDNA analysis can be used to monitor treatment response and to identify mechanisms of resistance in melanoma patients. CTC–derived xenografts can be used as a complement to improve personalized treatment selection.
Ogle, L.F. et al., 2016 [67]	69 hepatocellular carcinoma (HCC) patients	Blood CTCs	Multiparametric analysis, using flow cytometry, size, morphology and the investigation of DNA improved the detection of CTCs, which have predictive potential in HCC.
Wang, X. et al., 20167 [28]	200 non-small cell lung cancer (NSCLC) patients	Urine ctDNA	The *KRAS* mutational profile is highly concordant in urine and in corresponding tumor tissues. The longitudinal monitoring of mutations in transrenal DNA is helpful to stratify NSCLC patients according to predicted outcomes.
Okajima, W. et al., 2016 [64]	107 HCC patients	Plasma miRNA	Plasma miRNA-224 could be a sensitive biomarker to screen, monitor, and evaluate treatment in HCC.
Grimm, M. et al., 2016 [74]	92 oral squamous cell carcinoma (OSCC) patients	Blood Apo10 and transketolase like 1 (TKTL1) epitopes in monocytes	The significant decrease in epitope detection in monocytes (EDIM)-Apo10 and EDIM-TKTL1 scores after surgery suggest that these could be used as biomarkers to evaluate surgical resection and to monitor OSCC.
Malentacchi, F. et al., 2016 [62]	138 patients, including bladder (*n* = 93) and renal (*n* = 25) cancers, and prostate adenocarcinoma (*n* = 25)	Urinary carbonic anhydrase IX (CAIX) messenger RNA (mRNA)	The relative percentage of the full-length isoform CAIX mRNA in urine sediments could be used as a surrogate marker of CAIX expression in tumor tissues for kidney, prostate and bladder cancer diagnosis.
Lee, J.Y. et al., 2016 [42]	81 NSCLC patients	Plasma ctDNA	Analysis of *EGFR* mutations in plasma ctDNA is useful to monitor response and to promptly detect resistance in NSCLC patients treated with EGFR tyrosine kinase inhibitors (TKIs).
Gorges, T.M. et al., 2016 [11]	29 metastatic prostate cancer (PCa) patients	Blood CTCs	The detection of prostate specific membrane antigen (PSMA)-expressing CTCs could identify PCa patients that might benefit from targeted therapies and allow their monitorization.
Willms, A. et al., 2016 [66]	103 patients, including colorectal cancer (CRC) (*n* = 52), NSCLC (*n* = 40) and pancreas carcinoma (*n* = 11)	Serum tumor-associated microparticles (taMPS)	taMPs expressing epithelial cell adhesion molecule (EpCAM) and CD147 could be a promising biomarker for the diagnosis and monitoring of several neoplasias.
Endzeliņš, E. et al., 2017 [61]	50 PCa patients	Plasma circulating miRNAs and extracellular vesicle (EV)-associated miRNAs	miRNA profiles recovered from whole plasma and plasma extracellular vesicles (EVs) of PCa patients are different and have distinct diagnostic value.
Salvianti, F. et al., 2017 [21]	97 thyroid cancer patients	Plasma cell-free DNA (cfDNA)	The cfDNA integrity index [180/67 base pairs (bp)] is a promising biomarker for the diagnosis of thyroid carcinoma.
Xu, R-H. et al., 2017 [57]	1098 HCC patients	Plasma ctDNA	ctDNA methylation markers have utility for diagnosis, prognosis, and monitoring of HCC.
Cote, G.J. et al., 2017 [24]	75 medullary thyroid carcinoma patients	Plasma ctDNA	The detection of *RET* M918T mutations in plasma is highly specific but lacks sensitivity. The allelic fraction of ctDNA correlated with overall survival (OS) in thyroid carcinoma.
Insua, Y.V. et al., 2017 [9]	94 metastatic colorectal cancer (mCRC) patients	Blood CTCs	The gene expression panel used to detect CTCs was able to assess early treatment response, with improved efficiency in comparison to computed tomography (CT) scans in mCRC patients.
Xie, F. et al., 2018 [7]	150 NSCLC patients	Urine ctDNA	Urinary ctDNA informs about the tumor profile and serial monitoring could be used for prognosis of NSCLC.
Shoda, K. et al., 2017 [6]	60 gastric cancer patients	Plasma ctDNA	The copy number status of *HER2* is useful to monitor treatment efficacy in *HER2*(+) gastric cancer patients and to guide treatment decisions in patients showing a positive conversion of *HER2* status with recurrence.
Schøler, L.V. et al., 2017 [35]	45 CRC patients	Plasma ctDNA	Postoperative ctDNA is able to detect residual disease and early relapse in CRC.
Aaltonen, K.E. et al., 2017 [5]	36 metastatic breast cancer (mBC) patients	Blood CTCs	Gene expression alterations in CTCs could be related with treatment resistance and the characterization of these cells over time could help in treatment selection in mBC.
Barault, L. et al., 2018 [59]	182 mCRC patients	Plasma ctDNA	Methylation panels for ctDNA analysis can be used to monitor disease burden in mCRC patients.
Mastoraki, S. et al., 2018 [16]	122 mBC patients	Blood CTCs and plasma ctDNA	*ESR1* methylation status is highly concordant in CTCs and plasma ctDNA. *ESR1* methylation in CTCs was associated with lack of response to treatment in mBC patients.
Tjon-Kon-Fat, L-A. et al., 2018 [76]	50 castration resistant prostate cancer (CRPC) patients	Blood platelets	It is possible to find tumor-derived transcripts in platelets of CRPC patients that provide predictive information on treatment response and outcome.
Iwama, E. et al., 2017 [41]	35 lung adenocarcinoma patients	Plasma ctDNA	ctDNA analysis using droplet digital Polymerase Chain Reaction (ddPCR) is useful to predict treatment efficacy, while Next Generation Sequencing (NGS) can inform about resistance mechanisms in adenocarcinoma patients treated with afatinib.
Chen, S. et al., 2017 [47]	150 NSCLC patients	Urine ctDNA	Urinay cfDNA might be used as an alternative to tissue biopsies to determine *EGFR* status for diagnosis, prognosis and monitoring of NSCLC patients.
Shoda, K. et al., 2017 [51]	153 gastric cancer patients	Plasma circulating cell-free Epstein-Barr virus (cfEBV) DNA	Plasma cfEBV DNA might be useful to detect Epstein-Barr virus-associated gastric carcinoma (EBVaGC) and to monitor treatment response or disease progression in real-time.
He, J. et al., 2017 [12]	120 NSCLC patients	Blood CTCs and plasma ctDNA	CTCs and ctDNA capture the dynamic tumor profile during treatment and could complement current strategies for NSCLC management.
Chung, T.K.H. et al., 2017 [20]	117 cervical cancer patients	Plasma cfDNA	*PIK3CA* analysis in liquid biopsies shows promise to help in risk stratification of cervical cancer patients and to make informed treatment decisions.
García-Saenz, J.A. et al., 2017 [36]	49 breast cancer (BC) patients	Plasma ctDNA	Plasma ctDNA quantification has potential to monitor treatment outcomes, however, it might be limited by tumor heterogeneity and should be evaluated together with imaging data.
Christensen, E. et al., 2017 [37]	831 bladder cancer patients	Plasma and urine ctDNA	Monitoring *FGFR3* and *PIK3CA* mutations in urine and plasma samples of bladder cancer patients might be useful to monitor disease progression and recurrence.
Vidal, J. et al., 2017 [50]	115 mCRC patients	Plasma ctDNA	High concordance rates of *RAS* mutations in tumor tissue and ctDNA supports the use of liquid biopsies as a viable alternative to tissue biopsies for baseline diagnosis and to select candidates for anti-EGFR therapy.
Balaji, S.A. et al., 2018 [17]	180 patients, including lung (*n* = 9), breast (*n* = 42), colorectal (*n* = 22) and other cancers	Plasma ctDNA	ctDNA is a reliable marker in a large number of cancers and seems to have prognostic value at baseline.
Yang, Y-C. et al., 2018 [18]	47 CRC patients	Plasma ctDNA	Analysis of ctDNA provides additional clinical information regarding the tumor profile and could aid in early diagnosis and prognosis of CRC patients.
Qi, L-N. et al., 2018 [8]	112 HCC patients	Blood CTCs	CTCs are markers for early diagnosis and predictors of early recurrence. Epithelial-to-mesenchymal transition (EMT) and CTC release seem to be related to the overexpression of *BCAT1.*
Keup, C. et al. 2018 [10]	35 mBC patients	Blood CTCs and plasma EVs	EVs and CTCs display different mRNA profiles and might have potential to monitor therapy in mBC patients.
Almodovar, K. et al., 2018 [31]	27 small-cell lung cancer (SCLC) patients	Plasma ctDNA	ctDNA is a useful tool to monitor disease during treatment and to detect relapse prior to conventional imaging in SCLC.
Kodahl, A.R. et al., 2018 [34]	66 mBC patients	Serum ctDNA	The detection of *PIK3CA* mutations in tumor tissue and serum ctDNA is highly concordant. Detection of ctDNA *PIK3CA* mutations might complement imaging methods to follow treatment response in mBC.
Thomsen, C.B. et al. 2018 [3]	138 mCRC patients	Plasma ctDNA	Changes in ctDNA levels are related to progression risk during first line chemotherapy in *RAS/RAF* mutated mCRC patients.
Song, T. et al., 2018 [39]	150 mCRC patients	Urine cfDNA	There is a good concordance in DNA profiles of urine and tumor tissues. Monitoring total urine cfDNA levels could be used as a complement to mutation profiling, allowing to predict early treatment response and to identify mCRC patients at high risk.
Wang, D-S. et al., 2019 [43]	78 gastric cancer patients	Plasma ctDNA	Longitudinal sequencing of ctDNA is useful to monitor treatment of *HER2*(+) gastric cancer patients and to detect alterations driving resistance.
Khan, K.H. et al., 2018 (NCT02994888) [44]	47 CRC patients	Plasma ctDNA	cfDNA analysis is able to detect *RAS* pathway alterations in CRC patients that are classified as wildtype according to tumor tissues. Combining serial analysis of cfDNA and mathematical modeling allows to quantitatively predict the time needed for progression.
Bohers, E. et al., 2018 (NCT02339805) [53]	30 diffuse large B-cell lymphoma (DLBCL) patients	Plasma ctDNA	Liquid biopsies allow to correctly genotype DLBCL. cfDNA analysis could be used for follow-up as a complement to Positron Emission Tomography (PET) scan imaging.
Gao, W. et al., 2018 [15]	143 lung cancer patients	Blood CTCs	The combined use of immunomagnetic beads and ddPCR allows to sensitively detect CTCs, which have diagnostic value and potential for prognosis and monitoring of lung cancer patients.
Guibert, N. et al., 2018 (NCT02827344) [13]	96 NSCLC patients	Blood CTCs	It is possible to detect programmed death ligand 1 (PD-L1) expression in CTCs of NSCLC patients. CTCs were more frequently PD-L1(+) than tumor tissues and PD-L1(+) CTCs were found in all patients at progression.
Boffa, D.J. et al., 2017 (NCT01830426) [73]	112 NSCLC patients	Blood CTCs	PD-L1 expression in peripheral blood cells of NSCLC patients is associated with worse survival.
Jensen, T.J. et al., 2019 [56]	44 patients, including NSCLC (*n* = 8), melanoma (*n* = 8), breast cancer (*n* = 4), and others	Plasma ctDNA	The evaluation of dynamic copy number variations (CNVs) in cfDNA could serve as an early indicator of immunotherapy response or progression in various cancers.
Hong, X. et al., 2018 [14]	49 melanoma patients	Blood CTCs	RNA-based scoring of CTCs allows to serially monitor melanoma patients treated with immune checkpoint inhibitors (ICIs) and is predictive of clinical outcome.
Xue, L. et al., 2018 [77]	402 NSCLC patients	Blood tumor educated platelets (TEPs)	TEP RNA biomarkers could help NSCLC diagnosis and facilitate early detection.
Avogbe, P.H. et al., 2019 [23]	143 urothelial cancer patients	Plasma and urine ctDNA, and DNA from urinary exfoliated cells	The identification of *TERT* promoter mutations in urinary DNA is a highly sensitive and specific method for urothelial cancer detection, exceeding the performance of urine cytology for the detection of low-grade cancer.
Cheng, T.H.T. et al., 2019 [58]	46 bladder cancer patients	Urine ctDNA	Methylation and copy number analysis of urinary cfDNA allows to detect bladder cancer, which could be valuable for diagnosis and monitoring of tumor burden.
Sinha, S. et al., 2019 [26]	39 CRC patients	Plasma cfDNA	cfDNA quantity and integrity index (265/80 bp) is able to distinguish stage IV mCRC patients from healthy controls and might be useful for treatment monitoring.
Tian, J. et al., 2019 [27]	57 cervical cancer patients	Plasma ctDNA	Targeted deep sequencing of cfDNA is useful to monitor treatment response and to predict progression in cervical cancer.
Akamatsu, H. et al., 2019 (WJOG8114LTR) [29]	57 NSCLC patients	Plasma ctDNA	Liquid biopsies are able to predict treatment efficacy and progression in part of *EGFR*-mutated NSCLC patients.
Fernandez-Garcia, D. et al., 2019 [30]	194 mBC patients	Plasma cfDNA and blood CTCs	Total cfDNA levels and CTC number are predictors of disease response and outcomes in mBC.
Bernard, V. et al., 2019 [32]	194 pancreatic adenocarcinoma patients	Plasma ctDNA and DNA in exosomes (exoDNA)	Longitudinal monitorization of ctDNA and exoDNA provides prognostic information, which could be useful for therapeutic stratification of adenocarcinoma patients.
Benešová, L. et al., 2019 [33]	47 mCRC patients	Plasma ctDNA	ctDNA is useful for the early detection of recurrence and to confirm surgery extent in mCRC.
Zedan, A.H. et al., 2019 [63]	149 PCa patients	Plasma miRNAs	The changing levels of miRNA-93 and miRNA-221 during follow-up reveal their potential role for PCa monitoring.
Lv, J. et al., 2019 [38]	673 nasopharyngeal carcinoma patients	Plasma cfEBV DNA	Longitudinal quantification of cfEBV DNA in nasopharyngeal carcinoma during treatment adds prognostic value and may be helpful to adapt treatments according to risk.
Braig, D. et al., 2019 [22]	64 soft tissue sarcoma (STS) patients	Plasma cfDNA	Quantification and fragmentation analysis of cfDNA can distinguish patients with myxoid sarcomas from patients in complete remission or healthy individuals. Genotyping of ctDNA has potential to monitor myxoid sarcoma patients and to detect minimal residual disease and recurrence.
Cheng, J. et al., 2019 [40]	40 NSCLC	Plasma ctDNA	ctDNA analysis using a gene panel to study commonly mutated genes in NSCLC advanced tumors allowed to detect mutations at diagnosis, to monitor response to treatment, and to find resistance mutations.
Bordi, R. et al., 2019 (NCT02474335) [46]	38 NSCLC	Plasma ctDNA	The analysis of ctDNA *EGFR* mutations plays a crucial role in prognosis of NSCLC.
Egyud, M. et al., 2019 [48]	38 esophageal carcinoma patients	Plasma ctDNA	Plasma ctDNA is detectable and correlates with disease burden in esophageal carcinoma. ctDNA could be used to monitor treatment response and recurrence.
Francaviglia, I. et al., 2019 [49]	100 NSCLC patients	Plasma ctDNA	ctDNA is useful to identify therapeutic targets, to monitor therapy and to find mechanisms of resistance in NSCLC.
Malczewska, A. et al., 2019 [4]	99 bronchopulmonary carcinoid tumor (BPC) patients and 101 patients with other lung neoplasias	Blood mRNA	Increased levels of target transcripts are indicative of lung neuroendocrine neoplasia. Gene expression was concordant in blood and matched tumor tissues and allowed to identify disease progression accurately.
Pizzi, M.P. et a., 2019 [54]	46 gastric adenocarcinoma patients	Plasma and gastric wash ctDNA	Gastric washes are a source of ctDNA and could be used to track mutations in gastric adenocarcinoma patients. Combined analysis of gastric washes and plasma increased sensitivity of ctDNA detection.
Herrmann, S. et al., 2019 [55]	34 CRC patients	Plasma ctDNA	The use of custom amplicon panels allows to detect relevant sets of ctDNA mutations and to monitor treatment response and development of resistance in CRC.
Miller, A.M. et al., 2019 [60]	85 glioma patients	Cerebrospinal fluid (CSF) ctDNA	ctDNA from CSF collected from glioma patients is able to represent the tumor profile and could be used to track tumor evolution.
Iwama, E. et al., 2020 [45]	100 NSCLC patients	Plasma ctDNA	The analysis of mutations in cfDNA is useful to predict efficacy and to monitor clonal evolution during EGFR TKI treatment in NSCLC.
Yu, H. et al., 2020 [52]	150 mCRC patients	Plasma and urine ctDNA	Both plasma and urine ctDNA genotyping might have clinical utility in mCRC, namely for monitoring and risk stratification.
Sol, N. et al., 2020 [78]	89 primary glioblastoma patients and 126 patients with one or multiple brain metastases [primary tumors include: NSCLC (*n* = 85); BC (*n* = 15); melanoma (*n* = 15); renal cell carcinoma (*n* = 7); and others]	Blood TEPs	TEP-spliced RNA profiles enable high-accuracy classification compared with TEP-spliced RNA profiles from asymptomatic healthy controls and patients with neuro-inflammatory or other (neuro)oncological conditions. TEPs profiles are dynamic, indicating that TEPs can be employed for blood-based therapy monitoring.

*Note*: BC–Breast Cancer; bp–Base Pair; BPC–Bronchopulmonary Carcinoid; CAIX–Carbonic Anhydrase IX; cfDNA–cell-free DNA; cfEBV–circulating cell-free Epstein-Barr Virus; CNVs–Copy Number Variations; CRC–Colorectal Cancer; CRPC–Castration Resistant Prostate Cancer; CSF–Cerebrospinal Fluid; CT–Computed Tomography; CTCs–Circulating Tumor Cells; ctDNA–circulating tumor DNA; ddPCR–droplet digital Polymerase Chain Reaction; DLBCL–Diffuse Large B-Cell Lymphoma; EBVaGC–EBV-associated Gastric Carcinoma; EDIM–Epitope Detection in Monocytes; EMT–Epithelial-to-Mesenchymal Transition; EpCAM–Epithelial Cell Adhesion Molecule; EVs–Extracellular Vesicles; exoDNA–exosomal DNA; HCC–Hepatocellular Carcinoma; ICI–Immune Checkpoint Inhibitor; mBC–metastatic Breast Cancer; mCRC–metastatic Colorectal Cancer; miRNA–microRNA; mRNA–messenger RNA; NGS–Next Generation Sequencing; NSCLC–Non-Small Cell Lung Cancer; OS–Overall Survival; OSCC–Oral Squamous Cell Carcinoma; PCa–Prostate Cancer; PD-L1–Programmed Death Ligand 1; PET–Positron Emission Tomography; PSMA–Prostate Specific Membrane Antigen; SCLC–Small-Cell Lung Cancer; STS–Soft Tissue Sarcoma; taMPs–tumor-associated Microparticles; TEPs–tumor educated platelets; TKI–Tyrosine Kinase Inhibitors; TKTL1–Transketolase Like 1.

**Table 2:** Clinical applications and limitations of different liquid biopsy analytes.

Liquid Biopsy Analytes	Clinical Applications						Limitations
	Aid Diagnosis	Tumor Profiling	Prognosis	Monitoring Treatment Response	Early Identification of Resistance Mechanisms	Early Detection of Relapse or Residual Disease	
**Cell-free DNA (cfDNA)**	[17,21,22,26,39,47,52]	[6,17,29,32,35,40,42,43,44,50,52,53,57]	[3,17,28,31,33,35,37,41,42,44,45,48,49,50,55,75]	[3,6,29,31,34,37,38,39,41,42,45,46,48,49,51,53,59]	[28,29,43,47,50]	[3,31,33,35,38,42,48,50,51,75]	Low concentrations and low sensitivity of detection [12,41,44,92]
							Lack of standardized preanalytical protocols [92]
**Circulating Tumor Cells (CTCs)**	[8,15]	[8,12,13,16,30]	[14,15,67,73]	[9,10,11,14,30,75]	[5,12,75]	[8]	Rare in circulation [5,12,16]
							Difficult and costly isolation [5,12,16,30]
**Circulating RNAs**	[4,62,64,65]	[4,61,64,65]	[65]	[4,63,64,65]		[4,64]	Techniques in early development [93]
							RNA instability [93]
							Difficult to detect low abundance RNAs [93]
**Extracellular Vesicles (EVs) and Tumor-Associated Microparticles (taMPs)**	[61,66]	[32]		[10,61,66]			Lack of standardized preanalytical protocols [61,92]
**Tumor Educated Platelets (TEPs)**	[77]	[78]	[76]	[78]			Techniques in early development [42,78]

## Data Availability

No new data were created or analyzed in this study. Data sharing is not applicable to this article.
